# Modelling the structures of G protein-coupled receptors aided by three-dimensional validation

**DOI:** 10.1186/1471-2105-9-S1-S14

**Published:** 2008-02-13

**Authors:** Siavoush Dastmalchi, W Bret Church, Michael B Morris

**Affiliations:** 1 School of Pharmacy, Tabriz University of Medical Sciences, Tabriz 51664, Iran; 2 Biotechnology Research Center, Tabriz University of Medical Sciences, Tabriz 51664, Iran; 3 Faculty of Pharmacy, University of Sydney, Sydney, NSW 2006, Australia; 4 School of Medical Sciences, Human Reproduction Unit, University of Sydney, Sydney, NSW 2006, Australia

## Abstract

**Background:**

G protein-coupled receptors (GPCRs) are abundant, activate complex signalling and represent the targets for up to ~60% of pharmaceuticals but there is a paucity of structural data. Bovine rhodopsin is the first GPCR for which high-resolution structures have been completed but significant variations in structure are likely to exist among the GPCRs. Because of this, considerable effort has been expended on developing *in silico *tools for refining structures of individual GPCRs. We have developed REPIMPS, a modification of the inverse-folding software Profiles-3D, to assess and predict the rotational orientation and vertical position of helices within the helix bundle of individual GPCRs. We highlight the value of the method by applying it to the Baldwin GPCR template but the method can, in principle, be applied to any low- or high-resolution membrane protein template or structure.

**Results:**

3D models were built for transmembrane helical segments of 493 GPCRs based on the Baldwin template, and the models were then scored using REPIMPS and Profiles-3D. The compatibility scores increased significantly using REPIMPS because it takes into account the physicochemical properties of the (lipid) environment surrounding the helix bundle. The arrangement of helices in the helix bundle of the 493 models was then altered systematically by rotating the individual helices. For most GPCRs in the set, changes in the rotational position of one or more helices resulted in significant improvement in the compatibility scores. In particular, for most GPCRs, a rotation of helix VII by 240–300° resulted in improved scores. Bovine rhodopsin modelled using this method showed 3.31 Å RMSD to its crystal structure for 198 C^α ^atom pairs, suggesting the utility of the method even when starting with idealised structures such as the Baldwin template.

**Conclusion:**

We have developed an *in silico *tool which can be used to test the validity of, and refine, models of GPCRs with respect to helix rotation and vertical position based on the physicochemical properties of amino acids and the surrounding environment. The method can be applied to any multi-pass membrane protein and potentially can be used in combination with other high-throughput methodologies to generate and refine models of membrane proteins.

## Background

G protein-coupled receptors (GPCRs) are a family of integral-membrane proteins (IMPs) that transduce chemical and optical signals through the cell membrane [1] leading to the activation of G proteins, which in turn trigger a wide range of biological events [2]. GPCRs share a conserved structure consisting of seven transmembrane α-helices, as determined by a variety of methodologies including electron cryo-microscopy [3,4] and X-ray diffraction [5,6]. Detailed structural knowledge of GPCR structure is of interest in part because they are prime targets for therapeutic agents [7]. A number of web sites provide theoretical models and other information on GPCRs. For example, at GPCRDB , diverse data on GPCRs, including close to 2000 structural models, have been collected and organized [8].

Several modelling approaches have been used to construct three-dimensional models of GPCRs and can be classified broadly into two categories: those using structural templates [9-11] and those using *de novo *approaches [12-15].

Low- and high-resolution structures of bacteriorhodopsin (BR) have been used as templates for modeling the structures of GPCRs because of the seven transmembrane regions and the similarity of mechanism of activation of BR to that of rhodopsin. However, there are many assumptions inherent in the use of BR as a template. For example, although BR and rhodopsin are activated by light, BR functions as a proton pump [16,17] whilst rhodopsin is coupled to a G protein [18]. The sequence identity between BR and rhodopsin is low (12.8%) and a comparison of the high-resolution structures of BR and rhodopsin reveals different helix bundle arrangements [19]. Modelling of GPCRs based on alignment with the structure of BR may, therefore, be error prone [20]. Nevertheless, many 3D models of GPCRs have been generated based on the structure of BR, such as those of the receptors for dopamine, adrenalin, serotonin, acetylcholine [21-23], vasopressin V2 [9], opioids [13,24], guanine nucleotide-binding regulatory protein [25], human thromboxane A2 [26], 5-HT_2B _[27] and galanin [10,28].

The crystal structure of bovine rhodopsin, solved to a resolution of 2.8 Å, represents the first high-resolution structure of a GPCR [5,6]. Since then, this crystal structure has been used as a template for modelling other GPCRs [29] on the basis that the structure of rhodopsin represents a consensus template.

Lower-resolution templates have also been used to model GPCRs. The template developed by Baldwin *et al. *based on the electron density map of frog rhodopsin [3,4] includes the C^α ^positions of the 7 transmembrane helices as well as their extensions beyond the membrane on both sides. The sequences of 493 GPCRs were then examined using a consensus approach, based on residue conservation and hydrophobicity analysis of amino acids, and projected into the plane of the membrane to postulate several structural features of the family, including the location of the transmembrane segments within a sequence, transmembrane lengths and extensions beyond the membrane, and orientations of the helices with respect to one another. Strahs and Weinstein have modelled opioid receptors using comparative and molecular dynamics studies in which the transmembrane helix bundles were assembled on this Baldwin template [11]. Luteinizing hormone [30], α_1b_-adrenergic [31] and type one thyrotropin-releasing hormone [32] receptors are among other GPCR models based on the Baldwin template. Rubenstein *et al. *studied the mechanism of activation for β_2_-adrenergic receptor using molecular dynamics techniques and a biophysical model based on the Baldwin template [33]. The template continues to be useful as a starting point for modeling GPCRs even with the release of the crystal structure of bovine rhodopsin (see [34] and references cited therein).

However, no single template appears to be appropriate for modeling the structures of all GPCRs. For example, the use of the high-resolution structure of rhodopsin as a template has recently been questioned: the model of CCK1 receptor built based on the rhodopsin structure was unable to reproduce the experimentally observed interactions between the ligand (CCK) and the receptor model in docking approaches [35]. Similarly, for the Baldwin template, 'conserved' residues for a particular GPCR are not always present and often there is no obvious cluster of hydrophobic residues on one side of the helices to help locate them either vertically with respect to the membrane or with respect to rotation. Thus, the available data suggest that the organization of the transmembrane components of GPCRs is dictated by more considerations than contained in the currently available templates, regardless of the resolution. This is not surprising, given that sequence conservation among GPCRs can be low, they have adapted to bind a large range of ligand types and sizes, and nonidealities in the structure of transmembrane segments such as kinks, unwindings and tightenings are likely to be, in many cases, GPCR-specific.

Several inverse-folding methodologies have been developed to model the three-dimensional structures of proteins. These methods are based on physicochemical, as opposed to sequence homology, considerations and use potential functions frequently involving pairwise amino-acid interaction, solvent exposure, and local secondary structure. Based on these criteria, the probability of finding specific residues in a particular class of environment can be estimated. The string of residues of the protein is thus converted to a string of environment classes from which compatible structures can be generated.

Reverse-environment prediction of IMP structure (REPIMPS) [36] is a modification of the Profiles-3D application, an inverse-folding methodology appropriate for water-soluble proteins [37,38]. The modification accounts for the fact that sidechains of many residues in IMPs are in contact with lipid rather than water. The correction ensures that lipid-exposed residues are appropriately classified with respect to their physicochemical environment. As a result, compatibility scores calculated using REPIMPS for IMPs whose structures have been solved improve significantly over those calculated using Profiles-3D, and there is a reduced possibility of rejecting a 3D model of an IMP because the presence of a lipid environment was not included [36]. REPIMPS has been used to locate the transmembrane segment in IMPs with a single transmembrane domain, has the potential to locate transmembrane segments in IMPs with multiple transmembrane domains, and can be used to assess if transmembrane segments are appropriately oriented with respect to the lipid environment and surrounding transmembrane domains [36].

We highlight the value of the REPIMPS method by applying it to models of GPCRs generated from an idealised template, the Baldwin template, to test the validity of, and refine, the models with respect to helix rotation and vertical position. The method can, in principle, be applied to any low- or high-resolution GPCR template or structure, or to any multi-pass membrane protein, and potentially can be used in combination with other high-throughput methodologies to generate and refine models of IMPs.

## Results

### Large-scale comparative modelling of GPCRs based on the Baldwin template and calculation of lipid-corrected compatibility scores and CAD values

Three-dimensional models were built for the 493 GPCRs in the database used by Baldwin *et al. *[3], which contains the coordinates of the C^α ^atoms predicted to be part of the transmembrane segments and their helical continuations at both sides of the membrane. Side-chain positions were refined as outlined in Methods.

**Figure 1:**
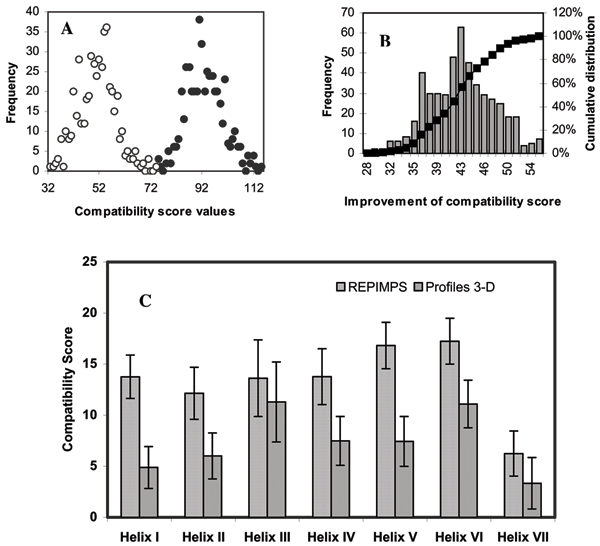
(**A**) Distribution of raw Profiles-3D (○) and lipid-corrected (●) compatibility scores (REPIMPS result) of 493 GPCR models. (**B**) Distribution of the improvement of the compatibility scores for the modelled GPCRs as a result of simulation of the presence of the hydrophobic lipid bilayer environment. Left y-axis represents the number of models, shown by bars, at each level of improvement and the right y-axis is the percentage of the cumulative distribution represented by the curve. (**C**) Average (± SD) of compatibility scores in the presence and absence of a lipid environment calculated by REPIMPS and Profiles-3D, respectively, for Helices I to VII of 493 models of GPCRs. Differences between compatibility scores calculated by REPIMPS and Profiles-3D methods are significant for all helices (p < 0.001).

For the 493 GPCR models, compatibility scores were calculated using Profiles-3D, which assumes an aqueous environment (Figure 1A). The compatibility scores were also calculated using REPIMPS [36], which assumes that atoms in contact with the membrane are in a hydrophobic environment (Figure 1A). The average lipid-corrected compatibility score using REPIMPS was 94 compared to an average score of 52 calculated using Profiles-3D. The level of improvement was not the same for all models. Figure 1B shows the distribution of the improvement in the compatibility scores for individual GPCRs calculated using REPIMPS *versus *the value calculated using Profiles-3D. Scores were also compared for individual helices as part of the whole model (Fig. 1C). For each of helices I–VII of the 493 GPCR models, the mean lipid-corrected compatibility scores, as calculated by REPIMPS, were significantly higher (p < 0.001) than the mean scores calculated using Profiles 3D, as determined by paired *t *test.

**Figure 2:**
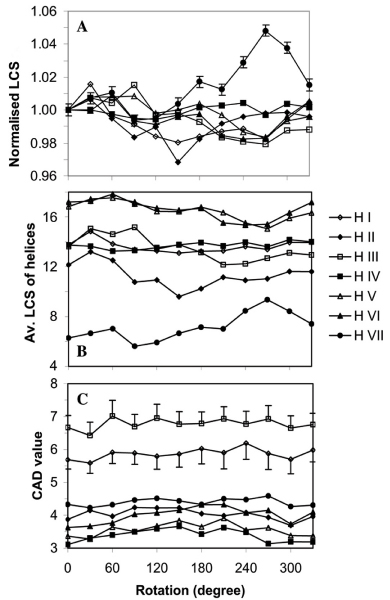
(**A**) Average of normalised lipid-corrected compatibility scores calculated using the REPIMPS method for the models of GPCRs plotted against the rotation of individual helices. Models of all 493 GPCRs used in this study were built based on the Baldwin template [3]. Changes were made to the models by rotating a single helix around the helix long axes every 30°. The average lipid-corrected compatibility score was calculated for all models at a particular rotational status and normalised for the average lipid-corrected compatibility score value at 0° rotational position. (**B**) Average of lipid-corrected compatibility scores calculated using the REPIMPS method for the helices of the models of GPCRs plotted against rotational orientation. Models of all 493 GPCRs used in this study were built based on the Baldwin template [3]. Changes were made to the models by rotating a single helix around the helix axes every 30°. The lipid-corrected compatibility score was calculated for a particular helix for all models at different rotational orientations and the average value was plotted. (**C**) Average of CAD scores for the modelled GPCRs *vs *rotation of individual helices. The CAD value was calculated for the comparison of a model GPCR in any particular rotational step of a helix with the next rotated neighbour. The CAD results for the same rotation change in all 493 GPCR models were averaged and plotted against the rotational degree. For example, Helix I of the model for a particular GPCR was rotated about its long axis from 0° to 30°, and then a CAD comparison was performed between them. The results of CAD calculations for this change in the models of all studied GPCRs were averaged and plotted at 0°.

In order to evaluate the arrangement of the helices in the helix bundle of the 493 models generated based on the Baldwin template, the model structures were altered systematically by rotating the individual helices one at the time by 30° about the helix long axes. For each rotation, the change in the lipid-corrected compatibility score was calculated using REPIMPS for all 493 GPCR models. The values were then averaged and normalized against the average score for the unchanged models (Figs 2A,B).

To analyse the degree of structural difference between consecutive pairs of models after rotation of each of the helices, contact area difference (CAD) values were calculated [39] and the average value for all models plotted against the rotation of individual helices (Fig. 2C). For example, helix I of the model for a particular GPCR was rotated from 0° to 30°, and then a CAD comparison was performed between them. The results of the CAD calculation for this change in the models of all 493 GPCRs were then averaged and plotted at 0°.

### REPIMPS-guided modelling of bovine rhodopsin and hGalR1

There is good agreement between the transmembrane regions of bovine rhodopsin determined from the crystal structure and the Baldwin template (Table 1): Superposition of the model for bovine rhodopsin derived from Baldwin template with the equivalent residues in the crystal structure gave an RMSD of 3.2 Å for the 198 C^α ^atoms, which suggests very similar arrangement of the helices [40]. For individual helices, the RMSDs were largely due to nonidealities (unwindings, tightenings and kinks), translation perpendicular to the membrane, and helix rotation up to ~30° [40]. Note that a small amphipathic helix is seen in the crystal structure following helix VII which is not present in the Baldwin template (Table 1). This helix is not predicted to be transmembrane but rather to lie on the cytoplasmic face of the membrane bilayer.

**Table 1:** Transmembrane helical segments of bovine rhodopsin and the number and position of transmembrane segments of hGalR1 predicted using different transmembrane prediction methods.

	***Method^b ^used for predictingTMS of hGalR1***							***TMS of bovine rhodopsin***	
			
**TMS**^*a*^	**DAS**	**HMMTOP**	**SOSUI**	**TMHMM**	**TopPred**	**TMPRED**	**Baldwin**^*d*^	**Crystal structure**^*c*^	**Baldwin**^*d*^
I	32–58	35–59	31–53	37–59	33–53	34–58	38–55	34–64	38–64
II	73–93	72–89	70–92	72–94	82–102	77–108	72–89	71–100	69–95
III	98–129	110–131	103–125	109–131	111–131	110–131	113–130	106–139	108–142
IV	153–166	152–171	151–173	151–173	151–171	152–172	155–168	150–173	151–175
V	201–221	202–221	201–223	205–227	201–221	199–221	203–219	200–225	204–233
VI	248–262	246–263	245–267	248–270	245–265	245–263	249–266	247–277	245–274
VII	281–286	290–307	271–293	285–307	268–288	265–288	289–304	285–309	288–311
VIII					286–306			311–320	

^*a*^Transmembrane segment.

^*b*^Transmembrane prediction methods used from their web sites and can be accessed from .

^*c*^TMS of bovine rhodopsin based on the crystal structure (pdb code 1F88  A).

^*d*^Transmembrane regions proposed by Baldwin *et al. *[3].

The strong similarity between the crystal structure of bovine rhodopsin and the model derived from the Baldwin template suggests the template remains a useful starting point for further refinement of the structures of individual GPCRs. Indeed, the presence of nonidealities present in the crystal structure may be, in some cases a disadvantage since these are likely to be GPCR-specific.

We examined several *in silico *tools used to predict the position and number of transmembrane segments of IMPs and compared the results when these tools were applied to the sequence for the GPCR, hGalR1 (Table 1). The exact locations, length and number of transmembrane segments predicted by the different tools varied. For example, TopPred predicted 8 transmembrane segments for hGalR1 and the predicted location of Helix VII using the various methods was, in some cases, mutually exclusive. The hydrophobicity and hydrophobicity moments of the transmembrane segments of hGalR1 proposed by Baldwin *et al. *[3] were also calculated (Table 2) [41]. The values of the moments were small, and consistent with values estimated for other IMPs [41].

**Table 2:** Average hydrophobicity and hydrophobicity moment of the transmembrane helices (I–VII) of hGalR1 based on the sequence alignment proposed by Baldwin *et al. *[3].

TMS^*a*^	Number of residues	Average hydrophobicity^*b*^	Hydrophobic moment^*c*^	Alpha phase^*d*^
I	27	0.68	0.06	239
II	27	0.38	0.18	157
III	35	0.47	0.17	17
IV	25	0.37	0.13	222
V	30	0.44	0.21	115
VI	30	0.38	0.09	339
VII	24	0.22	0.25	198

^*a*^Transmembrane segment.

^*b*^The hydrophobicity scale used in these calculations was the consensus scale of Eisenberg *et al. *[41].

^*c*^Mean vector sum of the hydrophobicities of the side chains of the helix.

^*d*^The angle of the moment from the first residue in the window, in the direction in which the α-helix turns.

Next, we used REPIMPS to consider a series of possible alignments of just the transmembrane segments of hGalR1 mapped onto the Baldwin template. The sequences used for the model building cover the sequence alignment postulated by Baldwin and four alternatives deviating from the Baldwin alignment by frameshifting the sequence by up to two positions in either direction, as shown for Helix I of hGalR1 in Table 3. This effectively generated models in which the helices were rotated along the helix axis and translated up and/or down relative to the other helices in the bundle. Using all possible combinations of the different sequences defined for the seven helices of hGalR1, a total of 78,125 models were generated.

**Table 3:** Sequences of Helix I used for building models of hGalR1. Sequence number 3 is the sequence of the transmembrane region proposed by Baldwin *et al. *[3].

Sequence identifier	Sequence
1	ENFVTLVVFGLIFALGVLGNSLVITVL
2	NFVTLVVFGLIFALGVLGNSLVITVLA
3	FVTLVVFGLIFALGVLGNSLVITVLAR
4	VTLVVFGLIFALGVLGNSLVITVLARS
5	TLVVFGLIFALGVLGNSLVITVLARSK

**Figure 3:**
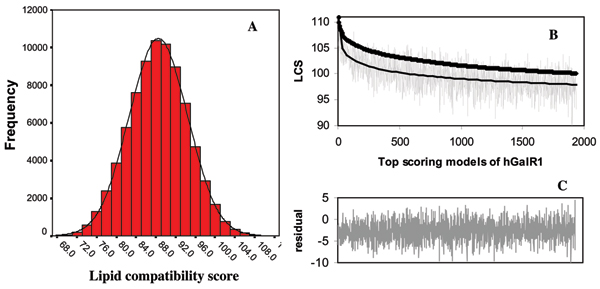
**(A) **Distribution of lipid compatibility scores calculated using REPIMPS for all 78,125 different models of hGalR1. The mean lipid-corrected compatibility score was 88.5. Top scoring models with lipid-corrected compatibility score greater than 100.13 (mean lipid-corrected compatibility score + 2 SD) were subjected to further structure refinement. Structural refinement was also performed on the Baldwin model of hGalR1 despite its low lipid-corrected compatibility score of 85.5.**(B) **Recalculation of lipid-corrected compatibility scores for the top-scoring models after structural refinement is shown in gray. The best fit line for the lipid-corrected compatibility scores of the refined models (thin line) are compared to the best fit line for the lipid-corrected compatibility scores before structural refinement (thick line). **(C) **The residual differences of the lipid-corrected compatibility scores before and after the structural refinement are shown. The lipid-corrected compatibility score generally decreased after structural refinement by a mean value of 2.6.

The lipid-corrected compatibility score for the Baldwin consensus model of hGalR1, as determined by REPIMPS, was 85.5. Fig. 3A shows the distribution of scores for all 78,125 models. The mean score was 88.5 ± 5.9σ. 1934 models had scores greater than two standard deviations above this mean (values > 100.13) and were subjected to further structure refinement by searching for energetically favourable rotamers for the side chains. Fig. 3B compares the lipid-corrected compatibility scores for these models before and after structure refinement. On average, structure refinement reduced the scores by a mean value of 2.6 (Fig. 3C). The sequences of the transmembrane helices for the model of hGalR1 with the highest lipid-corrected compatibility score following structure refinement are shown in Table 4 and compared with sequences derived from the Baldwin template for this receptor.

**Table 4:** Comparison of the sequences defining the transmembrane helices of pairs of models for bovine rhodopsin and hGalR1. The models of bovine rhodopsin and hGalR1 built using the group A sequences onto the Baldwin template give the lipid-corrected compatibility scores of 107.76 and 110.0, respectively. According to the REPIMPS method these are the best models for bovine rhodopsin and hGalR1 among 78,125 models built for each of these GPCRs. Group B sequences are those derived from alignment of sequences of 493 GPCRs proposed by Baldwin *et al. *[3]. The lipid-corrected compatibility score for bovine rhodopsin and hGalR1 modelled using group B sequences were 85.6 and 89.8, respectively.

Helix	Alignment	Sequences for bovine rhodopsin	Sequences for hGalR1
I	A	^37^**FSML**AAYMFLLIMLGFPINFLT*LYVTV*^63^	^35^**VTLV**VFGLIFALGVLGNSLVIT*VLARS*^61^
	B	^38^**SMLA**AYMFLLIMLGFPINFLTL*YVTVQ*^64^	^34^**FVTL**VVFGLIFALGVLGNSLVI*TVLAR*^60^
			
II	A	^70^*TPLNY*ILLNLAVADLFMVFGGFT**TTLY**^96^	^67^*RSTTN*LFILNLSIADLAYLLFCI**PFQA**^93^
	B	^69^*RTPLN*YILLNLAVADLFMVFGGF**TTTL**^95^	^67^*RSTTN*LFILNLSIADLAYLLFCI**PFQA**^93^
			
III	A	^106^**GPTGCNL**EGFFATLGGEIALWSLVV*LAIERYVVVC*^140^	^106^**FICKFIH**YFFTVSMLVSIFTLAAMS*VDRYVAIVHS*^140^
	B	^108^**TGCNLEG**FFATLGGEIALWSLVVLA*IERYVVVCKP*^142^	^106^**FICKFIH**YFFTVSMLVSIFTLAAMS*VDRYVAIVHS*^140^
			
IV	A	^152^*HAIMG*VAFTWVMALACAAPPL**VGWS**^176^	^148^*VSRNA*LLGVGCIWALSIAMAS**PVAY**^172^
	B	^151^*NHAIM*GVAFTWVMALACAAPP**LVGW**^175^	^150^*RNALL*GVGCIWALSIAMASPV**AYHQ**^174^
			
V	A	^202^**SFV**IYMFVVHFIIPLIVIFFC*YGQLVFTVK*^231^	^199^**AYV**VCTFVFGYLLPLLLICFC*YAKVLNHLH*^228^
	B	^204^**VIY**MFVVHFIIPLIVIFFCYG*QLVFTVKEA*^233^	^201^**VVC**TFVFGYLLPLLLICFCYA*KVLNHLHKK*^230^
			
VI	A	^247^*EKEVTRMVIIM*VIAFLICWLPYAGVAF**YIF**^276^	^240^*ASKKKTAQTV*LVVVVVFGISWLPHHII**HLW**^269^
	B	^245^*KAEKEVTRMVI*IMVIAFLICWLPYAGV**AFY**^274^	^240^*ASKKKTAQTV*LVVVVVFGISWLPHHII**HLW**^269^
			
VII	A	^286^**IFMT**IPAFFAKTSAVYNPVI*YIMM*^309^	^284^**FRIT**AHCLAYSNSSVNPIIY*AFLS*^307^
	B	^288^**MTIP**AFFAKTSAVYNPVIYI*MMNK*^311^	^285^**RITA**HCLAYSNSSVNPIIYA*FLSE*^308^

Residues in **bold **and *italic *are helical extracellular and intracellular residues, respectively. Underlined residues are exposed to lipid molecules. In order to calculate total compatibility scores for the whole model, lipid-based environment correction using REPIMPS was applied just to the intramembranous residues.

The model building method and identification of the transmembrane helices using the abovementioned procedures were also applied to bovine rhodopsin, the first GPCR for which there is a crystal structure. Using REPIMPS, the Baldwin model of this protein had a lipid corrected compatibility score of 85.6 and was not among the top scoring models, whilst the best model amongst the 78,125 models generated had a REPIMPS score of 107.8 and showed deviations from the model proposed by Baldwin (Table 4).

## Discussion

GPCRs are signalling molecules that traverse the cell membrane with seven helices in an anticlockwise progression as viewed from outside the cell. Though much evidence suggested this overall architecture [3,4], it was only with the first reported crystal structure of a GPCR that this was confirmed [5,6]. GPCRs bind ligands ranging from small molecules to large proteins, indicating that details of their architecture must deviate. Furthermore, additional factors that affect the structure and function of GPCRs, such as dimerisation and interactions with associated proteins, have been reported [42,43]. The overall result is that no single *in vivo *mechanism of a GPCR has been fully characterised at the structural level.

GPCRs are considered non-standard proteins based on the most applicable methods of structure determination [44], and so it is not expected that high-resolution structural data will accrue rapidly for this class of protein in the near future. For this reason, comparative protein modelling methods, which assume that a single template structure is appropriate for all members of a family, remain an important approach in modelling the structures of GPCRs and indeed all other families of IMP. However, these templates, whether high- or low-resolution, should only be regarded as a starting point for determining the unique structural properties of individual members within the family. Thus, *in silico *tools, such as those used to predict the location of transmembrane segments and to refine the structural features of the template, also remain an important feature of predictive modelling for IMPs.

We applied the REPIMPS methodology to a well-known template for GPCRs, the Baldwin template, to indicate that, for individual GPCRs, the rotational position of helices and their vertical positioning may differ significantly from the template. This *in silico *tool compares favourably with other tools in terms of predicting the location of transmembrane helices [36] (Tables 1, 4) and can, in principle, be applied to templates from any IMP family, including, for example, the high-resolution structure of the GPCR, bovine rhodopsin. The fact that so few high-resolution structures have been determined for GPCRs indicates that low-resolution templates, such as the Baldwin template, will continue to play a role in predictive modelling of individual GPCRs. In addition, we have shown previously that the crystal structure of bovine rhodopsin and the model of this protein derived from the Baldwin template show overall structural similarity (3.2 Å RMSD) [40]. Because of this, it is likely that both the high-resolution crystal structure of bovine rhodopsin and the Baldwin template remain valid starting points for building models of individual GPCRs with the aid of *in silico *tools such as REPIMPS.

Starting with either the crystal structure or the Baldwin template is likely to have its advantages and disadvantages. For example, the nearly idealised helices of the low-resolution template may prove useful in some respects since they lack the nonidealities of the crystal structure, such as localised unwindings, tightenings and kinks, which may well be GPCR-specific. Alternatively, it may prove useful in some cases to apply REPIMPS to models derived from the high-resolution structure in which the effects of the documented nonidealities and *in silico *mutations can be assessed.

### Improvement of the compatibility score of the Baldwin template for GPCRs using REPIMPS

We built 493 models of GPCRs based on the Baldwin template [3] and assessed them with the REPIMPS methodology, which unlike Profiles-3D from which it was derived, takes into consideration that sidechains of many residues in IMPs are in contact with lipid rather than water [36]. REPIMPS improved the average compatibility score for all 493 GPCR models to 94, compared to a value of 52 obtained with Profiles-3D (Figure 1A). The greatest improvement was seen for helices I and V which have a greater area exposed to the lipid membrane in the Baldwin template (Figure 1C). Similarly, the lowest improvement of compatibility scores was observed for helices III and VII which have the smallest area exposed to lipid. We previously demonstrated the existence of the correlation between area exposed to the solvent and the extent of the improvement of compatibility scores for a set of IMP structures [36].

The effects of the rotation of helices about their axis show that there are rotational steps for which the lipid-corrected compatibility score is significantly higher than that at the origin (zero rotation) (Figs 2A,B). A higher value for a rotated helix compared to that calculated for the Baldwin template at zero rotation is an indication that an alternate position for the helix is available which positions the side chains in a more compatible environment within the bilayer. This is most evident for helix VII, where it appears that for most GPCRs a rotation of 240–300° relative to the Baldwin template position is the preferred orientation. Alternatively, this may not be the consequence of misorientation of helix VII in the Baldwin template, but rather from nonidealities similar to those seen in the crystal structure of bovine rhodopsin [5,6]. Nevertheless, the REPIMPS approach suggests deficiencies in the Baldwin template purely using a molecular modelling approach based on placing IMPs in the correct (lipid) environment.

Most of the commonly used methods to evaluate the difference between a model and a reference structure are based on calculating RMSD values. However, the structural changes that we have applied to the models of GPCRs, namely the rotation of the helices, require the analysis of structural differences which are not dependent on the geometrical changes of the structure. For this reason, we used the CAD method [39] which measures a normalised sum of absolute differences of residue-residue contact surface areas calculated for a reference structure and a model.

The average of the CAD values were used to compare a pair of models, which differed by the rotation of a single helix by 30° about the helix axis. The CAD values for each helix for 493 models were plotted against the total rotation from the starting model as shown in Figure 2C. As is clear from the figure, rotation of the helices about the helix axis did not produce major fluctuations in the values. The maximum CAD value was ~7, seen for rotation of helix III. This is in the range observed for the differences between different models of a protein derived from structures solved using NMR techniques [39]. Because the relevance of the absolute values of the CAD method is difficult to determine, we were most interested relative changes. The calculations suggested there were no rotations that produced unrealistic changes.

For a more reasonable assessment of the differences between the models as the result of rotation of the helices, the CAD values were calculated for just that part of the model in which changes were made. In the case of bovine rhodopsin, the model was truncated to contain just the helix being rotated as well as those helices which are in contact with the rotated helix. In this way the average CAD value for helix I increased from 6.1 ± 0.3 to 9.4 ± 0.7. In a similar way, the CAD value as a result of rotation of helix III increased from 6.9 ± 0.3 to 9.5 ± 0.3. These higher CAD values are also in the range that one can expect for the differences between the models of a protein built based on the data from NMR spectroscopy. Overall, the process of rotating helices in GPCRs does not greatly affect CAD values. This provides justification for rotating helices and using REPIMPS to assess the quality of models and the appropriate orientation of helices.

### Modelling of bovine rhodopsin and hGalR1 based on the Baldwin template using REPIMPS and different alignments of transmembrane segments

We generated 78,125 models for both bovine rhodopsin and hGalR1 built using 5 different threads of sequences originating from each of the transmembrane regions. The models were then scored using the REPIMPS algorithm.

Figure 3A shows the distribution of the lipid-corrected compatibility scores for the generated models of hGalR1. A total of 1934 models with the score greater than the mean lipid-corrected compatibility score plus two standard deviations (>100.13) were subjected to more structure refinement by searching energetically more favourable rotamers for the side chains. The model representing the alignment derived using the Baldwin template was also included in the structure refinement step despite its low lipid-corrected compatibility score of 85.5. The sequences of the helices with the highest lipid-corrected compatibility score obtained by REPIMPS are shown in Table 4 (set A) along with the sequences for hGalR1 derived from the Baldwin template (set B). The two sets are identical for helices II, III and VI. Helix VII shows a one residue shift, while Helices I, IV and V are different by a two residue shift.

The validity of the models could be further tested by experimental means such as site-directed mutagenesis. There is evidence that Helix III [5,6], residues at the top of Helices IV and VII [45] and residues His^264 ^and His^267 ^in Helix VI [10,28] are important for galanin binding. Both the model generated directly from the Baldwin template and the 'refined' model generating using REPIMPS have the His residues positioned inside the helix bundle.

With respect to bovine rhodopsin, the REPIMPS approach identified a set of sequences for the transmembrane segments that are different from those for the model generated from the Baldwin template (Table 4). The REPIMPS-based model built from these sequences shows an RMSD of 3.31 Å to the crystal structure for 198 C^α ^atom pairs of the residues indicated in Table 4. This RMSD value is comparable to that obtained for the model of rhodopsin proposed by Shacham (2.9 Å) [46], Baldwin (3.2 Å) [40], and Yarov-Yarovoy (3.8 Å for 91 residues) [15]. In our model Lys^296 ^is moved 2.9 Å down in the helix axis and faces toward the binding pocket of the retinal molecule created by helices 3–7. In the Baldwin model, Lys^296 ^is facing helix 1, in a direction opposite to the binding pocket. The differences between our model and the crystal structure of bovine rhodopsin may be indicative of deficiencies in our method. However, the crystal structure represents just one form (the inactive form) of this receptor [47].

## Conclusion

REPIMPS can be used as an *in silico *tool to assist in the modelling positional features of transmembrane segments of IMPs. The method can, in principle, be applied to any template for GPCRs as well as templates for other families of IMP. Here, we have applied REPIMPS to the Baldwin template of GPCRs and shown that, individually and collectively, vertical positioning and rotational orientation of the transmembrane helices can differ significantly from the template.

## Methods

### GPCR sequences, 3D models and programs

Calculations were performed on a Silicon Graphics O2 workstation (SGI, Mountain View, CA, USA) using the InsightII molecular modelling package (v98.0, Molecular Simulations, San Diego, CA, USA, now available from Accelrys, San Diego, CA, USA). The Baldwin model of bovine rhodopsin, consisting C^α ^atoms in transmembrane helices and their extra-membrane extensions and the sequences of the predicted transmembrane regions of 493 GPCRs aligned based on the known GPCRs footprint residues, were obtained from Dr J. Baldwin [3]. Additional File 1 contains the details of the GPCRs modelled in this study. The crystallographic structure of rhodopsin was obtained from the Protein Data Bank at the Research Collaboratory for Structural Bioinformatics [48]. As an alternative to the RMSD comparison, the ICMlite program (v 2.8 2000, MolSoft L.L.C., La Jolla, CA) was used to calculate contact area difference (CAD) [39] between a pair of models and/or structures of the receptors. Secondary structure conformations were identified using the Kabsch-Sander method [49]. Use of the Profiles-3D program and REPIMPS method were as described previously [36]. Briefly, for IMPs a significant proportion of the residues are in contact with lipids of membrane. Thus, by considering the surrounding apolar environment, the correct values of the area of the sidechain buried away from the aqueous phase (A) and the area in contact with polar atoms (F) were calculated by modifying the Profiles-3D program. By using the F, A and local secondary structure of each residue located within the membrane of an IMP, the appropriate environmental class for each residue from the 18 environmental classes [38,50] is assigned, and accordingly the appropriate compatibility score for the residue is registered. The compatibility score for a protein/model structure or any part of the structure is the sum of the compatibility scores for the comprising residues.

### Automated comparative modelling of GPCRs based on the Baldwin C^α ^template for bovine rhodopsin

Model-building functions were written in Unix: transmembrane sequences of all 493 GPCRs were extracted sequentially from the files holding sequence alignments, resulting in a sequence file with seven lines corresponding to seven transmembrane segments for each GPCR. The sequence file was automatically used to build a model based on the C^α ^template for the transmembrane helices of bovine rhodopsin postulated by Baldwin *et al. *[3]. In this model-building procedure, a polyalanine polypeptide which was created based on the coordinates of the C^α ^atoms in the Baldwin model was used as the template for modelling the 493 GPCRs using the PROTEIN/BACKBONE command in InsightII. Side chains were positioned using the rotamer library [51] starting with bulky side chains: Residues Trp, Tyr, Phe, Ile, Met and Val, in that order, were considered to have moving side chains, and then the side chain rotamer search for remaining residues was applied. The 'best' rotamer was selected for the first residue in the list based on energy criteria (i.e., the lowest energy). Then, the best rotamer was selected for the next moving side chain, and so on. A cycle was defined as one complete pass through the list. The search terminated when the energy changed ≤0.05 kcal/mol from one cycle to the next, as defined by the CONVERGENCE parameter. Usually 3–4 cycles of rotamer search and energy calculations were required.

### Calculating lipid corrected compatibility scores (using REPIMPS) and CAD values

After all 493 models were built, their self compatibility scores calculated by Profiles-3D and their lipid-corrected compatibility scores were calculated using REPIMPS [36]. For each model, using the InsightII command line, each helix was subjected to 12 fixed rotations of 30° about the helix long axis using the automated Unix script. At each rotation, the side chains were repositioned according to the procedure outlined above and self compatibility and lipid-corrected compatibility scores recalculated. In all, 41,412 model structures were generated.

As part of the evaluation of differences between a pair of models or structures of the same receptor protein, we used ICMlite to calculate the CAD value to measure geometrical differences between two different conformations of the same molecule. CAD, as opposed to RMSD, is contact based and can measure the difference between 3D models with a wide range of accuracy [39]. We used this method to assess the difference between models before and after a single step of rotation of an individual helix. For example, the model generated after a 30° rotation of Helix I about its long axis was compared with the original model in which Helix I was not rotated. Unix and ICM language scripts were used to automate the calculation procedure. For bovine rhodopsin, the CAD calculation was also carried out using rotational steps of 20° and 10° intervals. In addition, for bovine rhodopsin, the CAD values as the result of rotation of Helices I and III were recalculated in a different way by ignoring all other helices except those in contact with the helix under rotation; i.e., Helices II and VII for Helix I, and the Helices II and IV in the case of Helix III.

### Modelling bovine rhodopsin and hGalR1 based on the Baldwin template and verification of the models using REPIMPS

#### Transmembrane segments of bovine rhodopsin and hGalR1

Transmembrane segments of bovine rhodopsin (shown in Table 1) are based on the assignment indicated in the Protein Databank (RCSB) for the 1F88  A crystal structure and transmembrane segments proposed by Baldwin. Transmembrane segments of hGalR1 were predicted using the methods listed in Table 1. These methods were used from their web interfaces accepting the default settings for all the parameters. The results of predictions of transmembrane segments were compared with that postulated by Baldwin *et al. *[3].

#### Model-building procedure and REPIMPS calculation

The following procedure explaining the method in the case of hGalR1 is the same as that used for bovine rhodopsin modelling. For each helix, five different sequences were used to build the helix. These five sequences were taken from same region of hGalR1 and they had the same length. For example, Helix I was represented by five sequences, each consists of 27 residues, with the first sequence starting at Glu^32 ^and ending at Leu^58^, the second sequence starting at Asn^33 ^and ending at Ala^59^, the third sequence starting at Phe^34 ^and ending at Arg^60^, etc. The third sequence in each set of five was the same as the sequence proposed by Baldwin *et al. *[3] for that helix. Note that for the first sequence, for example, residues 32–58 were given the same C^α ^coordinates as residues 34–60 of the third sequence. Effectively this led to having five helices of the same length and coordinates but different in residue composition, from which only one was used in each cycle of model building. In each cycle of building the model of hGalR1, one helix was selected from each of seven sets of helices to build a complete seven-helix bundle model which had the same C^α ^coordinates as the Baldwin template for GPCRs. Side-chain refinement was not included in the procedure. The lipid-compatibility score of the generated model was calculated based on the REPIMPS method. This cycle was repeated in an automated manner until all 78,125 combinations of the helices were used.

#### Refinement of the models of bovine rhodopsin and hGalR1

The models with a lipid-corrected compatibility score greater than two standard deviations above the mean lipid-corrected compatibility score calculated for all 78,125 models were selected for further refinements. Side chains were positioned in the energetically most favourable state by searching a side-chain rotamer library and calculation of the energy for the models as outlined above.

#### Hydrophobicity-moment calculation

The hydrophobicity moments for the transmembrane segments were calculated using the Moment program [52]. The numerical values of the hydrophobicities used in these calculations were from the consensus scale of Eisenberg *et al. *[52], which have been normalised so that the mean value of the hydrophobicities was zero with standard deviation of unity [53].

## List of abbreviations

REPIMPS: Reverse-Environment Prediction of Integral Membrane Protein Structure

CAD: Contact Area Difference

RMSD: Root Mean Square Difference

## Competing interests

The authors declare that they have no competing interests.

## Authors' contributions

SD was responsible for the original concept and design, performing calculations and drafting as well as refinements of the manuscript; WBC was responsible for the original concept of the calculations, as well as scripting and testing, and then refinements of the manuscript; MBM was involved in conceptual improvements of the calculations, manuscript refinement and experimentalist perspective on the biochemistry. All authors read and approved the final manuscript.

## Supplementary Material

Additional file 1 **Sequences information for G protein-coupled receptors**. This table contains the names and UniProtKB/Swiss-Prot ID tags of the GPCRs modelled in this study. Click here for file
