# Supplementary material for: Modelling the structures of G protein-coupled receptors aided by three-dimensional validation
Source: BMC Bioinformatics. 2008 Feb 13;9(Suppl 1):S14. doi: 10.1186/1471-2105-9-S1-S14 (PMC2259415; doi:10.1186/1471-2105-9-S1-S14)
Supplement: Additional file 1 — Sequences information for G protein-coupled receptors. This table contains the names and UniProtKB/Swiss-Prot ID tags of the GPCRs modelled in this study. [file 1471-2105-9-S1-S14-S1.doc]

Additional Table 1: Sequences of G protein-coupled receptors included in the current study

| **Ligand/receptor: species** | **UniProtKB/Swiss-Prot ID** |
| --- | --- |
| A. Cationic amines |  |
| Acetylcholine m5:man, rat | P08912, P08911 |
| m3:man, rat, pig, cow, chk | P20309, P08483, P11483, P41984, P49578 |
| m1:man, rat, pig, mse | P11229, P08482, P04761, P12657 |
| m :drosophila | P16395 |
| m4:man, rat, mse, chk | P08173, P08485, P32211, P17200 |
| m2:man, rat, pig, chk | P08172, P10980, P06199, P30372 |
| 5-Hydroxytryptamine 5HT2B:rat, mse, man | P30994, Q02152, P41595 |
| 5HT2C:rat, man, mse | P08909, P28335, P34968 |
| 5HT2A:rat, chns-hms, man, mse, pig, macac | P14842, P18599, P28223, P35363, P50129, P50128 |
| 5HT5A:rat, mse, man | P35364, P30966, P47898 |
| 5HT5B:rat, mse | P35365, P31387 |
| 5HT1F:man, rat, mse | P30939, P30940, Q02284 |
| 5HT1E:man | P28566 |
| 5HT1D:man, dog, rat, rab | P28221, P11614, P28565, P49145 |
| 5HT1B:man, rat, mse, opossum, chnes-hms, rab | P28222, P28564, P28334, P35404, P46636, P49144 |
| 5HT1A:man, rat | P08908, P19327 |
| 5HT:great pond snail | Q25414 |
| 5HT 2a, 2b:drosophila | P28285, P28286 |
| 5HT1:drosophila | P20905 |
| 5HT6:rat, man | P31388, P50406 |
| 5HT7:man, mse, rat, gn-pg | P34969, P32304, P32305, P50407 |
| Octopamine type-1 drosophila | P22270 |
| Histamine H2:rat, dog, man, gn-pg | P25102, P17124, P25021, P47747 |
| H1:cow, rat, gn-pg, man | P30546, P31390, P31389, P35367 |
| Dopamine D2:man, rat, cow, xenops | P14416, P13953, P20288, P24628 |
| D3:rat, mse, man | P19020, P30728, P35462 |
| D4:man, rat, mse | P21917, P30729, P51436 |
| D1A:man, rat, gldfsh, xenops, opossum, pig | P21728, P18901, P35406, P42289, P42288, P50130 |
| D1B:man, rat \ D1B:xenops; D1C:xenops; D1 tilapia | P21918, P25115 \ P42290; P42291; P47800 |
| D :drosophila | P41596 |
| Noradrenaline Alpha-2b:man, rat, mse | P18089, P19328, P30545 |
| Alpha-2c:man, rat, mse, opossum | P18825 P35369, P22086, Q01337, P35405 |
| Alpha-2a:man, rat, pig, mse | P08913, P22909, P18871, Q01338 |
| Alpha-2:gldfsh | P32251 |
| Alpha-1a:rat, man | P23944, P25100 |
| Alpha-1b:rat, gldn-hms, man | P15823, P18841, P35368 |
| Alpha-1c:cow, man, rat | P18130, P35348, P43140 |
| Beta-2:man, rat, mse, gldn-hms | P07550, P10608, P18762, P04274 |
| Beta-1:man, rat, mse, macac \ beta-1:turkey | P08588, P18090, P34971, P47899 \ P07700 |
| Beta-3:rat, mse | P26255, P25962 |
| Beta-3:man, cow | P13945, P46626 |
| Beta-4:turkey | P43141 |
| B. Proteins |  |
| Glycoprotein hormones :- |  |
| LH-CG:man, rat, pig, mse | P22888, P16235, P16582, P30730 |
| TSH:man, rat, dog, mse | P16473, P21463, P14763, P47750 |
| FSH:man, rat, macac, cow, sheep, horse, pig | P23945, P20395, P32212, P35376, P35379, P47799, P49059 |
| Unknown:sea anemone | P35409 |
| Unknown:great pond snail | P46023 |
| C5a anaphylatoxin:man, mse, dog | P21730, P30993, P30992 |
| Chemokines : MIP1a-RL1:mse | P51676 |
| CC-CKR1:man, mse | P32246, P51675 |
| CC-CKR3:man, mse | P51677, P51678 |
| CC-CKR4:man, mse | P51679, P51680 |
| CC-CKR2:man; CKR5:man, mse; CKRY:mse | P41597; P51681, P51682; P51683 |
| Interleukin-8 type A:man, rab | P25024, P21109 |
| Interleukin-8 type B:man, mse, rab, rat | P25025, P35343, P35344, P35407 |
| Unknown LCR1:man, cow | P30991, P25930 |
| Unknown BLR1:man, mse, rat | P32302, Q04683, P34997 |
| Unknown GPR2:man | P46092 |
| Unknown EBI-1:man, mse | P32248, P47774 |
| Unknown PPR1:cow | P35350 |
| Unknown GPR5:man | P46094 |
| Unknown V28:man; RBS11:rat | P49238; P35411 |
| C. Peptides |  |
| Formyl-methionyl peptide FPR3:man | P25089 |
| FPR2:man | P25090 |
| FPR1:man(R98, R26), rab, mse | P21462, Q05394, P33766 |
| Opioid peptide Mu-:rat, man, mse | P33535, P35372, P42866 |
| Kappa-:mse, rat, man, gn-pg | P33534, P34975, P41145, P41144 |
| Delta-:mse, rat, man | P32300, P33533, P41143 |
| ORL1:hum, rat, mse, gn-pg | P41146, P35370, P35377, P47748 |
| Somatostatin SS1:man, mse, rat | P30872, P30873, P28646 |
| SS4:rat, hum, mse | P30937, P31391, P49660 |
| SS2:man, mse, rat, cow, pig | P30874, P30875, P30680, P34993, P34994 |
| SS5:rat, man; SSX:man | P30938, P35346; P34988 |
| SS3:mse, rat, man | P30935, P30936, P32745 |
| Bradykinin B2:rat, mse \ B2:man | P25023, P32299 \ P30411 |
| B1:hum, rab | P46663, P48748 |
| Endothelin ET-a:man, cow, rat | P25101, P21450, P26684 |
| ET-b:man, rat, cow, pig, mse | P24530, P21451, P28088, P35463, P48302 |
| ET-c:xenops | P32940 |
| Angiotensin II AT1:cow, man, pig, rab, dog, rat, mse | P25104, P30556, P30555, P34976, P43240, P25095, P29754 |
| AT1b:rat, mse | P29089, P29755 |
| AT:turkey | P33396 |
| AT-R2:xenops | P35373 |
| AT2:rat, mse, man | P35351, P35374, P50052 |
| Neuropeptide Y Y1:man, rat, mse \ Y1:xenops | P25929, P21555, Q04573 \ P34992 |
| NY2:man | P49146 |
| NY4:man | P50391 |
| NY5:rat | Q63634 |
| NPY:drosophila | P25931 |
| Neuromedin B BB1:rat, man | P24053, P28336 |
| Gastrin releasing peptide BB2:mse, man | P21729, P30550 |
| Bombesin BB3:man, gn-pg \ BB4:toad | P32247, P35371 \ P47751 |
| Tachykinins NK3:man, rat, mse \ unknown:man | P29371, P16177, P47937 \ P30098 |
| NK2:man, rat, cow, mse, gldn-hms | P21452, P16610, P05363, P30549, P51144 |
| NK1:man, gn-pg, rat, mse | P25103, P30547, P14600, P30548 |
| Melanocortins : |  |
| Melanocyte-stimulating hormone:man | Q01726 |
| Melanocyte-stimulating hormone:mse | Q01727 |
| Melanocyte-stimulating hormone:cow | P47798 |
| Adrenocorticotropic hormone:man, cow | Q01718, P34974 |
| MC3:man, rat, mse | P41968, P32244, P33033 |
| MC4:man | P32245 |
| MC5:mse, rat \ sheep, man | P41149, P35345 \ P41983, P33032 |
| Vasopressin V2:man, rat, pig, cow | P30518, Q00788, P32307, P48044 |
| Vasopressin V1a:rat, man, sheep | P30560, P37288, P48043 |
| Vasopressin V1b:man, rat | P47901, P48974 |
| Oxytocin:man, pig | P30559, P32306 |
| Vasotocin:teleost fish | Q90352 |
| GnRH:man, rat, mse, sheep, cow, pig | P30968, P30969, Q01776, P32237, P32236, P49922 |
| Thrombin:chnes-hms, rat, mse \ man | Q00991, P26824, P30558 \ P25116 |
| Thrombin:xenops | P47749 |
| Neurotensin:rat, man | P20789, P30989 |
| TSH releasing hormone:mse, rat, man | P21761, Q01717, P34981 |
| Cholecystokinin CCK-A:rat, man | P30551, P32238 |
| CCK/gastrin CCK-B:rat, dog, hum, africn-rat, rab | P30553, P30552, P32239, P30796, P46627 |
| Galanin:man | P47211 |
| D. Unknown ligands, probably peptides |  |
| APJ:man | P35414 |
| EBI-2:man | P32249 |
| NKD:drosophila | P30974 |
| DTKR:drosophila | P30975 |
| GIR:mse | P30731 |
| C38C10.1:c.elegans | Q03566 |
| YQH2:c.elegans | Q09502 |
| YR13:c.elegans | Q09638 |
| GPR1:rat, man | P46090, P46091 |
| G10D:rat, mse | P31392, P43142 |
| RDC1:dog, man | P11613, P25106 |
| SENR(GPRE/14):rat | P48041(P49684) |
| GPRA/10:man | P49683 |
| GPR7:man | P48145 |
| GPR8:man | P48146 |
| GPR9:man | P49682 |
| GCY4:man | P51684 |
| GCY6:man | P51685 |
| GC96:man | P51686 |
| GPRF/15:man | P49685 |
| GPR4:man, pig | P46093, P50132 |
| PAR-2:mse | P55086 |
| CNL3/GPR6:man, rat | P46095, P51651 |
| gpcr21/GPR3:man, mse | P46089, P35413 |
| R334/gpcr01/GPRC:man, mse, rat | P47775, P35412, P30951 |
| E. Viral receptors for unknown ligands |  |
| UL33:human cytomegalovirus | P16849 |
| US27:human cytomegalovirus | P09703 |
| US28:human cytomegalovirus | P09704 |
| ECRF3:herpesvirus saimiri | Q01035 |
| VK02:swinepox virus | Q08520 |
| F. Other molecules |  |
| Adenine nucleotides : ATP P2Y1:chk, turkey | P34996, P49652 |
| P2Y:cow, man, rat, mse | P48042, P47900, P49651, P49650 |
| Uridine P2Y4:man | P51582 |
| P2U:rat, mse | P41232, P35383 |
| Unknown 6H1:chk; GCRT:man | P32250; P43657 |
| Melatonin mel1c:xenops, chk | P49219, P49288 |
| mel1a:man, sheep, chk | P48039, P48040, P49285 |
| mel1b:man | P49286 |
| Adenosine A1:dog, cow, rat, man, rab, gn-pg, chk | P11616, P28190, P25099, P30542, P34970, P47745, P49892 |
| A2A:dog, man, rat, gn-pg | P11617, P29274, P30543, P46616 |
| A2B:rat, man | P29276, P29275 |
| A3:man, sheep \ A3:rat | P33765, P35342 \ P28647 |
| Prostanoids : EP3:mse, rat, man, cow, rab, pig | P30557, P34980, P43115, P34979, P46069, P50131 |
| EP1:mse, man | P35375, P34995 |
| EP4:mse, man, rat | P32240, P35408, P43114 |
| EP2:man | P43116 |
| IP2:man, mse, rat | P43119, P43252, P43253 |
| FP2:man, cow, rat, mse | P43088, P37289, P43118, P43117 |
| Thromboxane A2:mse, rat \ man | P30987, P34978 \ P21731 |
| Platelet activating factor:man, gn-pg \ rat | P25105, P21556 \ P46002 |
| Cannabinoid CB1:rat, man, mse | P20272, P21554, P47746 |
| Cannabinoid CB2:man, mse | P34972, P47936 |
| Unknown EDG-2:sheep | P46628 |
| Unknown EDG-1:man, rat | P21453, P48303 |
| Unknown HM74:man | P49019 |
| Unknown H218/AGR16:rat | P47752 |
| G. Light |  |
| Red pigment:gldfsh, cvfsh, | P32313, P22332 |
| Red pigment:man, mrmost, chk, chmln; green:man | P04000, P34989, P22329, P41592; P04001 |
| Pigment 521:gecko | P35358 |
| Green pigments 1, 2:cvfsh | P22330, P22331 |
| Green pigment:chk; blue pigment:chamln | P28683; P51471 |
| Green pigment 1, 2:gldfsh; 3:cvfsh | P32311, P32312; P51474 |
| Pigment 467:gecko | P35357 |
| Rh:cvfsh, gldfsh, carp; UV:zebrafish | P41590, P32309, P51488; P35359 |
| Rh:sandgoby, | P35403 |
| Rh:lamprey | P22671 |
| Rh:frog, bullfrog, chamln, alligator, xenops, chk | P31355, P51470, P41591, P52202, P29403, P22328 |
| Rh:man, cow, sheep, mse, chnes-hms, dog, rab, | P08100, P02699, P02700, P15409, P28681, P32308, P49912, |
| macac, rat | Q28886, P51489 |
| Pineal opsin:chk, pigeon | P51475, P51476 |
| Blue pigment:chk | P28682 |
| Blue pigment:gldfsh | P32310 |
| Blue pigment:cvfsh | P51472 |
| Blue pigment:man, cow, mse; violet:chk, xenops | P03999, P51490, P51491; P28684, P51473 |
| Insect opsin4:drome, drovi, drops | P08255, P17646, P29404 |
| Insect opsin3:drome, drops | P04950, P28680 |
| Insect opsin2:drome, drops | P08099, P28679 |
| Insect opsin1:drome, drops \ blowfly | P06002, P28678 \ P22269 |
| Opsin:mantis | P35362 |
| Rh:carpenter ant; desert ant | Q17292, Q17296 |
| Rh:crayfish | P35356 |
| Rh A, B:horseshoe crab | P35360, P35361 |
| Rh1, 2:crab | Q25157, Q25158 |
